# Live Bird Markets of Bangladesh: H9N2 Viruses and the Near Absence of Highly Pathogenic H5N1 Influenza

**DOI:** 10.1371/journal.pone.0019311

**Published:** 2011-04-26

**Authors:** Nicholas J. Negovetich, Mohammed M. Feeroz, Lisa Jones-Engel, David Walker, S. M. Rabiul Alam, Kamrul Hasan, Patrick Seiler, Angie Ferguson, Kim Friedman, Subrata Barman, John Franks, Jasmine Turner, Scott Krauss, Richard J. Webby, Robert G. Webster

**Affiliations:** 1 St. Jude Children's Research Hospital, Memphis, Tennessee, United States of America; 2 Jahangirnagar University, Dhaka, People's Republic of Bangladesh; 3 University of Washington, Seattle, Washington, United States of America; Duke-National University of Singapore Graduate Medical School, Singapore

## Abstract

Avian influenza surveillance in Bangladesh has been passive, relying on poultry farmers to report suspected outbreaks of highly pathogenic H5N1 influenza. Here, the results of an active surveillance effort focusing on the live-bird markets are presented. Prevalence of influenza infection in the birds of the live bird markets is 23.0%, which is similar to that in poultry markets in other countries. Nearly all of the isolates (94%) were of the non-pathogenic H9N2 subtype, but viruses of the H1N2, H1N3, H3N6, H4N2, H5N1, and H10N7 subtypes were also observed. The highly pathogenic H5N1-subtype virus was observed at extremely low prevalence in the surveillance samples (0.08%), and we suggest that the current risk of infection for humans in the retail poultry markets in Bangladesh is negligible. However, the high prevalence of the H9 subtype and its potential for interaction with the highly pathogenic H5N1-subtype, i.e., reassortment and attenuation of host morbidity, highlight the importance of active surveillance of the poultry markets.

## Introduction

Influenza A is a negative-strand RNA virus that uses aquatic birds as reservoir hosts [1], [2] and is classified by the surface proteins hemagglutinin (HA) and neuraminidase (NA). There are 16 HA and 9 NA subtypes, but not all combinations have been recovered from aquatic birds [2]. In the aquatic bird host, replication primarily occurs in the intestinal tract [3]. As such, most avian influenza viruses (AIV) cause only limited morbidity and mortality in birds. However, several subtypes, including H5N1, H7N7, H9N2, can infect the lungs and respiratory tracts of birds (and other animals), resulting in significant levels of disease and death [4], [5], [6], [7], [8], [9].

The first human outbreak of the highly pathogenic (HP) H5N1 subtype occurred in Hong Kong in 1997 [4], [9]. Since then, surveillance efforts to detect the HP H5N1-subtype virus in birds have increased in Asia. HP H5N1 has been detected in animals in Bangladesh, and in the nearby countries of Bhutan, China, India, Myanmar, Nepal, and Thailand. Human infections with HP H5N1 have been reported in Bangladesh and Myanmar (http://www.oie.int/eng/info_ev/en_AI_avianinfluenza.htm). As of February 24, 2011, there have been 384 reported outbreaks of HP H5N1 subtype at either backyard or commercial farms in 49 of the 64 districts of Bangladesh [10].

In addition to HP H5N1, the H9N2 subtype has been implicated in contributing to the influenza outbreak in 1997. Extensive surveillance efforts to discover the source of the Hong Kong pandemic revealed that influenza of subtype H9N2 was being isolated from chickens at the same markets that had HP H5N1-positive chickens [8]. Although the overall prevalence of the H9N2 subtype was low (4.4%), one market exhibited an unusually high prevalence (36.6%). During this outbreak, most chickens appeared healthy [8]. This observation and later experiments suggest that chickens that were previously infected by a H9N2-subtype virus may have been partially protected from the pathogenicity induced by a HP H5N1-subtype infection, which could have allowed the HP H5N1-subtype virus to ‘silently’ attain a prevalence where transmission to humans was probable [11], [12]. After the mass slaughter of poultry at these markets, HP H5N1 subtype was thought to be eradicated until it re-emerged in Hong Kong bird markets in 2001 [13]. Phylogenetic analysis later revealed that the HP H5N1 subtype was a reassortant virus that had obtained 6 internal gene segments from the H9N2-subtype virus A/Quail/Hong Kong/G1/97 (G1) [14] and the NA gene segment from the H6N1-subtype virus A/Teal/Hong Kong/W312/97 [15]. These H9N2-subtype viruses that contain gene segments similar to those of the HP H5N1 subtype have become established and are circulating in the poultry markets in Southeast Asia and the Middle East [16], [17], [18], [19], [20], [21]. Thus, the internal genes of the HP H5N1 virus have remained in circulation in the poultry.

Most of the information regarding influenza infection in Bangladesh has focused on passive surveillance of backyard or commercial farms [22], [23]. This report primarily details influenza infection in several live bird markets of Bangladesh because of the hypothesized role that live bird markets play on the epidemiology of AIV [8], [19]. Active surveillance efforts were concentrated on the retail markets, with farms, pet bird markets, and migratory birds also being sampled. H9N2-subtype influenza is circulating in the bird markets, and HP H5N1 subtype has been found, albeit at extremely low levels. These findings highlight the need for continued, active surveillance in poultry markets.

## Materials and Methods

### Sample collection

**Figure 1:**
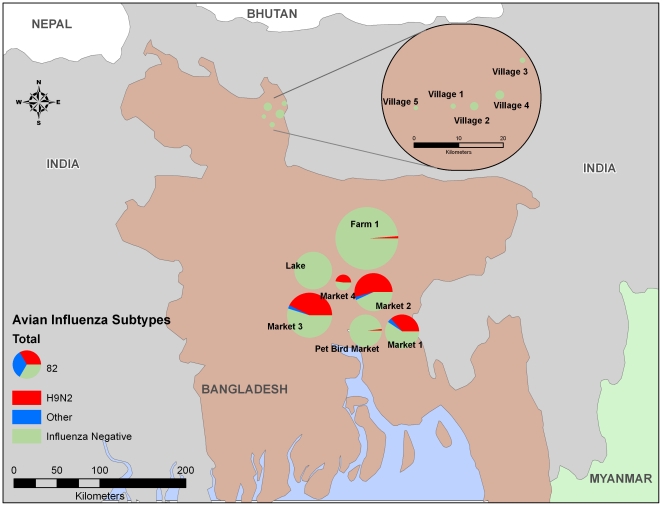
Map of Bangladesh that summarizes the surveillance data. A total of 1177 samples were tested for influenza A by virus isolation. The approximate locations of the sampling sites are presented on the map, with the pie chart corresponding to the relative proportion of total numbers of swabs in AI subtyped classes by study site. Samples testing positive for Newcastle disease virus were excluded from the analysis.

Active influenza surveillance was performed in Bangladesh from November 2008 through April 2009, and from December 2009 through July 2010 (Figure 1). Because AIV has a fecal-oral transmission route, samples were collected by taking oropharyngeal or cloacal swabs of birds (host samples) or by collecting from feces, fecal digestors, water troughs, and standing water (environmental samples). Each month, 300–600 total samples were collected across several locations. The primary sampling locations were retail markets in Dhaka (Market-1 to Market-4); one pet bird market, a layer farm (Farm-1), and a natural lake were also included. After news of an outbreak in 2009, surveillance was expanded to several villages in the area of the outbreak. The sampling sites in the expanded area were mostly backyard (domestic) flocks (Village-1 to Village-5), but several samples were also obtained from retail markets in 2 villages (Market-5 and Market-6) and from a poultry farm that culled their flock of chickens (Farm-2). None of the birds that were swabbed or observed near the sampling location exhibited signs of disease, and none of the sampling sites were reported as an outbreak area during the study.

### Sample screening

Samples were screened in 1 of 2 ways. Initially, all swab samples were injected into 10-day-old embryonated chicken eggs. After 72 hr of incubation at 35°C, eggs were chilled and harvested. Influenza-positive eggs were detected by testing for hemagglutination of 0.5% chicken erythrocytes according to standard procedures [24]. Shortly after the start of the surveillance program, real-time RT-PCR (rRT-PCR) was selected as an alternative to virus isolation in eggs to decrease the processing time of the samples. Viral RNA was extracted by using a KingFisher Flex Magnetic Particle Processor (ThermoFisher Scientific, Waltham, MA) and subjected to rRT-PCR using influenza A-specific primers and probes [25]. The reactions were performed in an ABI 7500 Fast Real-Time PCR machine (Applied Biosystems, Carlsbad, CA). All rRT-PCR-tested positive samples, and some rRT-PCR-tested negative samples, were injected into eggs to confirm the presence, or absence, of infectious virus. The percentages of positive samples reported in this manuscript exclude those samples that were subtyped as Newcastle disease virus (NDV) unless otherwise noted in the text. Differences in prevalence were assessed by using the proportions test in R version 2.11.1 (www.R-project.org).

### Subtyping of isolates

Where virus isolation was successful, sequence analysis [26] or hemagglutination inhibition (HI) assays [27] were performed to determine the subtype of influenza-positive samples. The Hartwell Center for Bioinformatics and Biotechnology at St. Jude Children's Research Hospital analyzed the sequences on Applied Biosystems' 3700 DNA analyzers by using BigDye Terminator (v. 3) chemistry and synthetic oligonucleotides. If cloacal and oropharyngeal swabs of the same host had more than one subtype of influenza, then the infection was identified as a mixed infection.

### Assessing pathogenicity

To assess the pathogenicity of the viruses, the intravenous pathogenicity index (IVPI) for several isolates was determined according to standards established by the World Health Organization [28]. Briefly, 0.1 mL of a 1∶10 dilution of infective egg chorioallantoic fluid in sterile PBS was intravenously injected into each of 10 six-week-old, specific pathogen-free (SPF) chickens. The birds were examined twice daily for 10 days, and individuals were scored on the basis of the observed morbidity (0 = normal, 1 = sick, 2 = severely sick, 3 = dead). Chickens were used in accordance with protocols approved by the Institutional Animal Care and Use Committee at St. Jude Children's Research Hospital (protocol 081). The study was approved by the Institutional Biosafety Committee at St. Jude Children's Research Hospital (#03A-137 and #02A-221).

## Results

### Influenza prevalence

A total of 5715 samples from several locations in Bangladesh were tested for AIV. The results of the virus isolation and rRT-PCR detection methods on identical samples were in agreement for 86.4% of the samples. Comparing the percent positive between the 2 methods is not meaningful because rRT-PCR was used to test 84.1% of the samples, yet virus isolation was performed on most of the suspected positives and only a small portion of negatives. Therefore, the percentage of infection as identified by virus isolation will be higher than expected if all of the samples had been tested by this method.

For some of the samples, cloacal and oropharyngeal swabs were collected from the same bird. The results of the matched swab samples agreed for 77.8% (n = 239) and 78.6% (n = 714) of the hosts when tested by the virus isolation and rRT-PCR detection methods, respectively. Overall, oropharyngeal swabs were more likely to test positive than cloacal swabs were regardless of detection method. Furthermore, this observation holds true when swabs were analyzed by sampling site (Table 1).

**Table 1:** Percentage of swab samples from hosts that tested positive for influenza A.

Site	Primary species*	% Virus isolation positive†			% rRT-PCR positive†		
		C	OP	Total	C	OP	Total
Farm-1	Ck	0 (35)	0 (35)	0 (38)	7.2 (111)	8.1 (111)	11.7 (111)
Farm-2	Pn				0 (4)	0 (4)	0 (4)
Lake	Dk	0 (3)	0 (3)	0 (3)	0 (3)	0 (3)	0 (3)
Market-1	Ck	15.8 (38)	36 (50)‡	32.2 (59)	10.6 (188)	15.4 (188)	22.6 (190)
Market-2	Ck	40 (60)	77.9 (77)‡	70.1 (87)	17.6 (227)	35.2 (227)‡	39.3 (229)
Market-3	Ck	12.8 (86)	52.1 (117)‡	51.7 (116)	15.8 (146)	40.3 (144)‡	44.3 (149)
Market-4	Ck	0 (21)	57.1 (21)	57.1 (21)			
Market-5	Ck				0 (3)	0 (3)	0 (3)
Market-6	Ck				0 (5)	0 (5)	0 (5)
Village-1	Ck	0 (2)	0 (1)	0 (3)	8.3 (12)	0 (12)	8.3 (12)
Village-2	Ck	0 (7)	0 (7)	0 (7)	0 (7)	0 (7)	0 (7)
Village-3	Ck	0 (2)	0 (3)	0 (3)	0 (5)	0 (5)	0 (5)
Village-4	Ck	0 (7)	0 (7)	0 (7)	0 (7)	0 (7)	0 (7)
VIllage-5	Ck/Dk	0 (2)	0 (1)	0 (2)	0 (5)	0 (5)	0 (5)
Total		15.6 (263)	46.9 (322)‡	43.9 (346)	12.7 (723)	24.4 (721)‡	29.2 (730)

*Primary species at each site. Species other than the primary species may have been sampled. Ck = Chicken, Dk = Duck, Pn = Pigeon.

† Percentage of cloacal and oropharyngeal swabs, or total hosts that tested positive. The number of tested swabs is listed in parentheses. The total percentage testing positive refers to individual birds regardless of the type of swab sample. C = Cloacal swab, OP = Oropharyngeal swab.

‡ Prevalence for the oropharyngeal swab was significantly higher (*P*<0.05) than the prevalence observed for the cloacal swabs. The proportions test was used to identify significant differences, and the test requires non-zero prevalence values for both cloacal and oropharyngeal swabs within a row. Comparison of values was performed for each of the detection methods.

Hosts were considered positive for infection if at least one of the host samples, i.e., oropharyngeal or cloacal swabs, tested positive by virus isolation or rRT-PCR (Table 2). The prevalence of influenza in hosts varied across the sampling sites, and analyses of prevalence revealed that differences exist across the sites for both virus isolation and rRT-PCR detection methods (p≤0.0001 and p≤0.00001, respectively). A significant difference in prevalence across the sites was also detected for the environmental samples (feces, fecal digestors, water, and water troughs) and when all samples, regardless of type, were tested by both detection methods (p≤0.00001 for all comparisons). In Dhaka, the overall prevalence varied by site, but this difference was mostly due to low prevalence at the Pet bird market, Farm-1, and the Lake. That is, prevalence was approximately equal at the markets (Table 2). Weighted average of prevalence for the markets in Dhaka was 47.3% and 23.0% for samples tested by virus isolation and rRT-PCR, respectively.

**Table 2:** Percentage of surveillance samples that tested positive for influenza A.

		Environment†		Host‡		Total§	
Site	Primary species*	% VI	% rRT-PCR	% VI	% rRT-PCR	% VI	% rRT-PCR
Farm-1	Ck	1.1 (366)^b^	3 (724)^c^	0 (38)	11.7 (111)^c^	1 (404)^c^	4.2 (835)^d^
Farm-2	Pn		0 (2)		0 (4)		0 (6)
Lake	Dk	0 (143)	1.7 (1453)^c^	0 (3)	0 (3)	0 (146)	1.7 (1456)^e^
Market-1	Ck	48.3 (58)^a^	40 (20)^a^	32.2 (59)^c^	22.6 (190)^b^	40.2 (117)^b^	24.3 (210)^b^
Market-2	Ck	36.7 (60)^a^	61.9 (21)^a^	70.1 (87)^a^	39.3 (229)^a^	56.5 (147)^a^	41.2 (250)^a^
Market-3	Ck	35.6 (90)^a^	8.4 (548)^b^	51.7 (116)^b^	44.3 (149)^a^	44.7 (206)a,b	16.1 (697)^c^
Market-4	Ck	0 (4)		57.1 (21)a,b		48 (25)a,b	
Market-5	Ck				0 (3)		0 (3)
Market-6	Ck				0 (5)		0 (5)
Pet bird market	Pt	1.9 (108)^b^	2.7 (590)^c^			1.9 (108)^c^	2.7 (590)d,e
Village-1	Ck			0 (3)	8.3 (12)	0 (3)	8.3 (12)
Village-2	Ck			0 (7)	0 (7)	0 (7)	0 (7)
Village-3	Ck		0 (1)	0 (3)	0 (5)	0 (3)	0 (6)
Village-4	Ck	0 (1)	0 (1)	0 (7)	0 (7)	0 (8)	0 (8)
Village-5	Ck/Dk			0 (2)	0 (5)	0 (2)	0 (5)
Total		10.6 (830)	3.9 (3360)	43.9 (346)	29.2 (730)	20.4 (1176)	8.4 (4090)

*Primary species at each site. Species other than the primary species may have been sampled. Ck = Chicken, Dk = Duck, Pn = Pigeon, Pt = Pet birds.

† Consists of fecal collections and samples from water troughs, standing water, and fecal digesters. Sample size is listed in parentheses.

‡ Reported as % positive for either a cloacal or oropharyngeal swab. Number of hosts that were tested is listed in parentheses.

§ Total number of samples tested irrespective of sample type. Sample size is listed in parentheses.

a–d Results of sequential proportions tests on the sampling sites in Dhaka. Atleast 1 positive sample was required for to site to be included in the analysis. Significant differences were not found between values with the same letter (*P*>0.05), but values with different letters were found to be significantly different (*P*<0.05). Sites with equal prevalences were combined and compared to the remaining sites to confirm that differences in prevalence exist. The statistical results are only valid when examining values within a column. Lower letters indicate higher prevalence values.

Nearly all of the host samples (84.3%) were collected from chickens (Phasianidae), the most abundant species at most of the sites. Exceptions include the lake, where the lesser whistling duck (Anatidae) is the primary inhabitant, and the pet bird market. The species list at the pet bird market includes species of the following: cockatiels (Cactuidae); doves (Columbidae); finches (Fringillidae); moorhens (Rallidae); mynah (Sturnidae); woodpecker (Picidae); love birds and parakeets (Psittacidae); munia and sparrows (Estrildidae); and pheasants and quail (Phasianidae). All birds in our dataset were grouped by family to increase the sample size of the host samples so that differences in prevalence of infection as detected by virus isolation could be examined. The major families that were infected included the Columbidae (11.4% prevalence, *n* = 114), Anatidae (25.0% prevalence, *n* = 24), and Phasianidae (33.2% prevalence, n = 754); prevalence differs by family (p≤0.0001).

### Influenza subtypes and pathogenicity

Subtyping of viruses by hemagglutination inhibition assays or by sequencing could only be performed on the 20.4% of samples that were influenza-positive by egg isolation. This represents 252 virus isolates from individual hosts or environmental samples (Table 3). Twelve isolates were subtyped as containing only NDV. Most of the influenza isolates were H9-subtype viruses (94.2%). The H9 subtype was identified at most of the retail markets (Figure 1). Based on the likelihood of obtaining the H9 subtype from rRT-PCR-positive host or environmental samples (76.3% of rRT-PCR positive samples were also virus isolation positive; 94.0% virus isolation positive isolates from the markets of Dhaka were H9-subtype viruses), the estimated prevalence of the H9-subtype in Dhaka is 16.5%. The H9-subtype isolates that were completely subtyped were all H9N2-subtype viruses. Other subtypes were observed in the markets including H1N2, H1N3, H3N6, H4N2, H5N1, and H10N7 (Table 3). The HA2 region of the H1-subtype viruses, which was used for subtyping by sequencing, is most similar (>90%) to that of avian H1 viruses and not to the pandemic H1-subtype viruses, with highest homology (94%) to A/goose/Italy/296426/2003 (H1N1). Although none of the birds that were sampled or in proximity to the sampling location were overtly sick, the IVPI was calculated for 5 isolates of the H9N2 subtype (A/Chicken/Bangladesh/559/2008, A/Environment/Bangladesh/600/2008, A/Environment/Bangladesh/907/2009, A/Environment/Bangladesh/5473/2010, and A/Environment/Bangladesh/5721/2010) and one H5N1-subtype isolate (A/Chicken/Bangladesh/828/2009). All of the H9N2-subtype isolates were non-pathogenic (IVPI = 0), but the H5N1-subtype virus was highly pathogenic (IVPI = 2.95).

**Table 3:** The number of isolates of each of the identified subtypes at the sampling sites.

Site	Primary species*	H1N2	H1N3	H3N6	H4N2	H5N1	H9N2	H10N7	Influenza negative†
Farm-1	Ck						4		400
Lake	Dk								146
Market-1	Ck	1	3	1			42		70
Market-2	Ck	1			1	2	79		64
Market-3	Ck					2	87	3	114
Market-4	Ck						12		13
Pet bird market	Pt						2		106
Village-1	Ck								3
Village-2	Ck								7
Village-3	Ck								3
Village-4	Ck								8
Village-5	Ck/Dk								2
Total		2	3	1	1	4	226	3	936

*Primary species at each site. Species other than the primary species may have been sampled. Ck = Chicken, Dk = Duck, Pt = Pet birds.

† Total number of samples (regardless of type) that were influenza negative by virus isolation.

### Serotyping

Seventeen H9N2-subtype isolates were tested using HI assays to determine the specific H9-antigenic group of the Bangladesh isolates (Table 4). Included in the analyses were chicken α-H9 sera that was generated during the IVPI experiments by using A/Chicken/Bangladesh/559/2008 (H9N2) and A/Environment/Bangladesh/600/2008 (H9N2). The isolates from Bangladesh reacted strongly to all of the anti-H9 sera. Most of the isolates reacted less strongly to the α-G1 sera than to the remaining antisera.

**Table 4:** Hemagglutination inhibitation titers.

Antigen		H9 Antisera*						
		a-559	a-600	a-G1	a-G9	a-Y280	a-HK/1073	a-Tk/MN
Bangladesh	A/Chicken/Bangladesh/559/2008	**10240**	10240	2560	10240	5120	10240	20480
	A/Environment/Bangladesh/600/2008	10240	**10240**	2560	5120	5120	10240	20480
	A/Chicken/Bangladesh/302/2008			640	5120	2560	2560	5120
	A,P/Chicken/Bangladesh/316/2008			640	2560	2560	5120	5120
	A/Chicken/Bangladesh/338/2008			640	2560	2560	2560	5120
	A/Chicken/Bangladesh/471/2008			1280	5120	5120	5120	5120
	A/Chicken/Bangladesh/501/2008			640	2560	1280	5120	5120
	A/Chicken/Bangladesh/511/2008			640	5120	2560	5120	5120
	A/Chicken/Bangladesh/537/2008			640	2560	1280	2560	5120
	A/Chicken/Bangladesh/567/2008			1280	10240	2560	5120	10240
	A/Environment/Bangladesh/583/2008			320	2560	1280	2560	5120
	A/ Environment /Bangladesh/5451/2010	2560	2560	320	1280	1280	2560	2560
	A/ Environment /Bangladesh/5452/2010	1280	2560	320	1280	1280	2560	2560
	A/ Environment /Bangladesh/5462/2010	1280	1280	160	1280	640	1280	2560
	A/ Environment /Bangladesh/5469/2010	1280	1280	160	1280	640	1280	2560
	A/ Environment /Bangladesh/5472/2010	1280	1280	160	1280	640	1280	2560
	A/ Environment /Bangladesh/5473/2010	1280	1280	160	640	640	1280	1280
Non-Bangladesh	A/Quail/Hong Kong/G1/97	640	640	**5120**	320	320	5120	5120
	A/Chicken/Hong Kong/G9/97	2560	5120	2560	**10240**	10240	5120	20480
	A/Duck/Hong Kong/Y280/97	640	640	320	2560	**5120**	1280	10240
	A/Hong Kong/1073/99	320	320	1280	320	320	**5120**	5120
	A/Turkey/Minnesota/38391-6/95	80	≤40	≤40	≤40	≤40	≤40	**2560**
	A/Chicken/Beijing/1/94	160	160	≤40	640	320	320	1280
	A/Quail/Dubai/301/2000†	1280	640	1280	1280	2560	5120	10240
	A/Chicken/Dubai/339/2001†	2560	1280	1280	2560	2560	5120	10240

*Antisera against the common H9 serotypes. The abbreviations are the isolate number of the antigen against which the sera were made. All are chicken antisera except α-A/Chicken/Hong Kong/G9/97 and α-A/Hong Kong/1073/99, which are goat or sheep hyperimmune antisera, respectively. Homologous sera are in boldface. Titers are expressed as the reciprocal of the last dilution that completely inhibited hemagglutination of 0.5% chicken erythrocytes.

† Isolates reported in Aamir et al. (2007).

## Discussion

Influenza surveillance in Bangladesh began in 2007 and was focused on the commercial and backyard farms [22], [23]. Here, we concentrate on the live bird markets because of the hypothesized role that live bird markets play on the epidemiology of AIV [8], [19]. An outbreak of AIV was reported in 2009 and surveillance was extended to villages in that area, but the primary focus remained on the markets.

Several conclusions can be drawn from this study. First, prevalence in the migratory waterfowl that visit the Lake is extremely low (1.7%), which suggests that AIV might not be maintained in birds at the lake ecosystem. Second, Farm-1 is a contaminated area, but the transmission within the flock appears to be at a minimum level as shown by the low prevalence at the site compared to that in the markets (rRT-PCR: 4.2% vs. 23.0%; Table 2). Third, although environmental samples collected near the birds (e.g., cockatiels, parrots, munia, quail) at pet bird markets tested positive for infection, only two samples were influenza A and the rest were subtyped as NDV. The 2 influenza-positive samples were subtyped as H9N2 and were collected from quail feces during the same visit to the pet bird market. Thus, the role of pet bird markets in perpetuating and spreading AIV remains unclear. Lastly, AIV is circulating in the 3 retail markets that were sampled in Dhaka. Chickens are the primary birds at these markets, but influenza has also been isolated from the ducks at all 3 locations. Therefore, there exists the possibility of interspecies transmission of influenza and an increased probability of reassortment between various lineages and subtypes. In fact, others have postulated that live bird markets are the ideal environment for influenza transmission because of the high density and variety of hosts within a localized area [19], [29], [30]. Indeed, reassortment of H9-subtype viruses in bird markets has been previously reported [18], [19], [20].

The H9N2-subtype viruses were primarily isolated from chickens. In contrast, all but 1 of the H1, H3, H4, and H10 subtypes were recovered from ducks; a single H1N2-subtype virus was isolated from a water trough in a chicken cage. The ducks could have obtained these infections at the market, but it is more likely that the birds were infected prior to arrival at the market and are contributing to the diversity of subtypes that exist in these locations [19]. This diversity increases the probability that reassortment could occur between influenza subtypes and produce a novel pathogenic strain. Of particular importance was the isolation of several H5N1-subtype viruses (clade 2.2), 2 of which were isolated from chickens in the same bird market in January 2009 and the remaining 2 were from fecal samples of ducks in a different poultry market in January 2010. These H5N1-subtype isolates exhibit a polybasic cleavage site (-PQGERRRKKRGLFG-) that is characteristic of highly pathogenic viruses. Moreover, one isolate (A/Chicken/Bangladesh/828/2009 [H5N1]) is also classified as highly pathogenic because of its IVPI. The isolation of H5N1-subtype viruses in the poultry markets could be related to outbreaks that were reported in commercial farms at approximately the same time. Specifically, an outbreak at a poultry farm in Mirpur, Dhaka, was reported on January 7, 2009 (http://www.oie.int/eng/info_ev/en_AI_avianinfluenza.htm), and HP H5N1-subtype viruses were isolated 2 days later from chickens at a poultry market in Dhaka. Although no outbreaks were reported at commercial poultry operations in the Dhaka district in Janurary 2010, an H5N1-subtype outbreak was reported at a farm in the Sirajgonji district, and another was reported in the Jaipurhat district (http://www.oie.int/eng/info_ev/en_AI_avianinfluenza.htm). Infected birds could have been transported to the market, but it is unknown if the infected birds were from commercial farms or from backyard flocks. Thus, the source of HP H5N1-subtype viruses in the bird markets cannot be determined. Interestingly, the chickens that were shedding the HP H5N1-subtype virus and the ducks from which the H5N1-infected fecal samples were collected were apparently healthy at the time of sampling, and H9N2-subtype viruses were isolated from other birds at the same markets. This raises an important question regarding the epidemiology of viruses of the H5N1 and H9N2 subtypes in the bird markets: are the H9N2-subtype viruses contributing to highly pathogenic H5N1-subtype virus outbreaks?

Before the H5N1-subtype outbreak in Hong Kong, no chickens and very few ducks tested positive for H5N1-subtype viruses, and H9N2-subtype viruses were only isolated from ducks in those markets [8]. Extensive surveillance during the outbreak in 1997 revealed high prevalence of the HP H5N1 subtype in apparently healthy chickens. This also corresponded with the surprising find of the H9N2 subtype in chickens, which had been reported only in ducks in the markets of Southeast Asia prior to the 1990's [8]. The prevalence of the H9N2 subtype in the Hong Kong markets in 1997 (4.4%) [8] was lower than its estimated prevalence in the Bangladesh retail markets (16.5%). Interestingly, a single retail market in Hong Kong in 1997 had an isolation rate of 36.6% for the H9N2 subtype, and the HP H5N1 subtype was isolated at the same market [8]. Later studies demonstrated that the H9N2 subtype might have allowed the HP H5N1 subtype to persist and circulate in poultry. Specifically, a previous H9N2-subtype infection could confer protection against an HP H5N1-subtype challenge [11], [12]. This may explain why the HP H5N1-infected chickens in the market were asymptomatic at the time of sample collection even though the virus killed all SPF chickens in the IVPI experiment by day 2 post-infection. Given that the primary influenza A subtype in poultry markets that we have been sampling in Bangladesh is H9N2, that HP H5N1-subtype outbreaks were reported in poultry in 2007 and continue today [22], and that HP H5N1-subtype viruses were isolated in the markets, Bangladesh could experience an outbreak similar to the Hong Kong 1997 outbreak.

Molecular analysis of the H9N2-subtype viruses that were in circulation in 1997 in Hong Kong suggest that H9N2 viruses have established a stable lineage in chickens [14], [31]. A single H9N2-subtype virus, A/Quail/Hong Kong/G1/97 (G1), was most similar to the HP H5N1 subtype from the 1997 outbreak in 6 of the 8 gene segments, and this lineage still circulates today [14], [32]. Serological evidence suggests that the H9N2-subtype isolates from Bangladesh are less like the G1-like reference virus, but the high degree of reactivity to the remaining reference sera obfuscates serological classification. The H9N2-subtype viruses from the United Arab Emirates exhibited a similar pattern of HI assay titers. In the original analysis, the authors concluded that these viruses were antigenically similar to the A/Duck/Hong Kong/Y280/97 (H9N2) virus (Y280 lineage), yet the results of phylogenetic characterization suggest that these viruses are genetically similar to the G1-like viruses [16]. Genetic characterization of the viruses from Bangladesh is in progress to examine the molecular evolution of the H9N2 subtypes in that country.

A higher percentage of oropharyngeal compared to cloacal swabs were positive for influenza infection. This observation raises concern regarding the mode of transmission for these viruses. Several H9N2-subtype viruses can transmit through direct contact with infected chickens and ferrets [33], [34]. It is this route of infection that likely produced the H9N2-subtype infections that have been reported in humans [6], [7], [22]. However, aerosol transmission has been observed in chickens infected with a Beijing/1-like virus (A/Chicken/Shanghai/F/98 [H9N2]) [33], and this could explain the high prevalence of the H9N2 subtype in the live-bird markets of Bangladesh. Aerosol transmission has not been observed between ferrets that were infected with H9N2-subtype viruses, suggesting that transmission between mammals is currently limited [34].

Influenza A of subtype H9N2 is established and circulating in poultry throughout the Middle East and Asia [16], [17], [18], [19], [20], [21]. Whereas previous studies in Bangladesh identified H9N2-subtype viruses on 3 farms [22], current surveillance efforts demonstrate that H9N2 is the primary subtype circulating in chickens at the retail markets. In the markets of Bangladesh, the H9N2 subtype has an estimated prevalence of 16.5%, which is higher than the reported prevalence of 7–8% in the live-bird markets in South Korea [18], [20]. Overall prevalence of the H9N2 subtype is lower in the markets of southern China from 2000–2005, but prevalence peaked at 13% in chickens, 22% in other minor poultry species, and 3% in ducks [35]. The H9N2 subtype has been isolated sporadically from the swine population throughout China [36], [37], [38]. The results of the phylogenetic analyses of the swine isolates suggest that these viruses are reassortants between H5- and H9-subtype viruses [36], [37]. To date, the H9N2-subtype reassortant viruses have not established a stable lineage in the swine population, and it is unknown if these reassortants are currently circulating in the live-bird markets.

The H9N2 subtype in the markets is of concern to humans because many isolates, including all of those sequenced in this study, possess the L226 mutation in the receptor binding pocket of the HA1 protein. This mutation confers a higher degree of specificity to sialic acid residues that contain the human-like α-2,6 linkage [39], and it has allowed the H9N2-subtype virus to grow in human airway epithelial cells [40] and be directly transmitted in ferrets [34]. The L226 mutation and the potential for aerosol transmission increases the risk of H9N2-subtype infection for humans that work in and visit the markets. Moreover, the H9N2 subtype may be modulating the morbidity and mortality that is associated with infection with the HP H5N1 subtype. Thus, early detection of the HP H5N1 subtype requires continuing surveillance efforts in the poultry markets.
